# Efficacy and Safety of Voriconazole for Difficult-to-Treat Distal Lateral Subungual Onychomycosis (DLSO)

**DOI:** 10.3390/pathogens14101049

**Published:** 2025-10-17

**Authors:** Aditya K. Gupta, Mesbah Talukder, Elizabeth A. Cooper, Lee Magal, Avner Shemer

**Affiliations:** 1 Division of Dermatology, Department of Medicine, University of Toronto, Toronto, ON M5S 1A8, Canada; 2 School of Pharmacy, BRAC University, Dhaka 1212, Bangladesh; mesbah.talukder@bracu.ac.bd; 3 Department of Physiology and Pharmacology, University of Western Ontario, London, ON N6A 5C1, Canada; elizzcooper@gmail.com; 4 Shemer Clinic, Netanya 4250602, Israel; leemagal@gmail.com; 5 Department of Dermatology, Sheba Medical Center, Tel-Hashomer, Ramat-Gan 52621, Israel; ashemer1@gmail.com; 6 Sackler Faculty of Medicine, Tel Aviv University, Tel Aviv 6997801, Israel

**Keywords:** distal lateral subungual onychomycosis (DLSO), voriconazole, refractory onychomycosis, terbinafine failure, clinical cure, mycological cure

## Abstract

This retrospective case series evaluated off-label voriconazole for distal lateral subungual onychomycosis (DLSO) unresponsive to standard therapy. Twenty-nine culture-confirmed patients who had failed terbinafine (250 mg daily × 12 weeks) and itraconazole pulses (200 mg twice daily for 1 week/month × 3) received voriconazole (200 mg twice on day 1, then 200 mg daily for 3–4 months). Assessments occurred at 2, 4, 6–9, and 12 months; the primary endpoint was combined clinical cure (≥90% nail clearance) plus mycological cure (negative KOH and culture) at 12 months. Intention-to-treat included 29 patients; per-protocol included 27 (two did not complete follow-up). In the per-protocol cohort, combined cure was 55.6% (15/27) and mycological cure 74.1% (20/27). Complete clinical cure occurred in 66.7% (18/27); 25.9% (7/27) improved markedly, 3.7% (1/27) mildly, and 3.7% (1/27) showed no improvement. Voriconazole was well tolerated and may be considered for DLSO refractory to terbinafine ± itraconazole. Antifungal stewardship remains essential.

## 1. Introduction

Onychomycosis commonly presents as distal lateral subungual onychomycosis (DLSO), a chronic infection that can cause pain, secondary bacterial infection, and reduced quality of life [1,2]. First-line therapy for mild disease typically involves topical agents such as efinaconazole, tavaborole, ciclopirox, or amorolfine, whereas oral antifungals (e.g., terbinafine, itraconazole, or fluconazole) are preferred for moderate-to-severe cases [3]. However, treatment outcomes are often suboptimal due to slow toenail growth, poor nail plate penetration, and prolonged treatment durations [4,5]. Randomized trials of efinaconazole 10% show mycologic cure rates ~53–55% and complete cure ~15–18% after 48–52 weeks, while tavaborole trials report lower complete cure (≈6.5–9.1%), underscoring variable topical efficacy [6,7,8]. An additional challenge is the growing prevalence of terbinafine resistance, commonly associated with mutations in the squalene epoxidase (SQLE) gene, prompting consideration of alternative therapies for recalcitrant onychomycosis [9,10].

Voriconazole, a broad-spectrum second-generation triazole antifungal agent, is approved for serious systemic mycoses [11,12]. However, it is not formally indicated for onychomycosis. Nonetheless, it may be used off-label in refractory cases [12]. Evidence for its efficacy in this context, however, remains sparse. We present a retrospective case series evaluating the clinical and mycological outcomes of voriconazole therapy in patients with treatment-refractory DLSO.

## 2. Methods

### 2.1. Study Design and Population

This retrospective case series evaluated 29 patients with distal lateral subungual onychomycosis (DLSO) treated with voriconazole. Of the 29 patients, two discontinued follow-up.

### 2.2. Treatment Protocol and Outcome Assessment

All patients with culture proven dermatophyte and clinical presentation of DLSO initially received terbinafine at a dose of 250 mg daily for 12 weeks. In the case of insufficient clinical improvement, the treatment was changed to itraconazole pulse therapy, consisting of 200 mg twice daily for one week per month, administered over three pulses. If this regimen also resulted in insufficient clinical improvement patients were offered voriconazole, 200 mg orally twice daily on the first day, followed by 200 mg orally once daily for 3–4 months. To assess the efficacy and safety of voriconazole treatment, patients were evaluated at 2, 4, 6–9, and 12 months after starting voriconazole treatment. Treatment was considered effective if, at 12 months from the start of voriconazole therapy, the patient had both clinical cure (at least 90% improvement in the involved nail plate area) and mycological cure (negative KOH microscopy and culture).

### 2.3. Statistical Method

This retrospective study included data from 27 patients who completed the treatment course (number of patients who started therapy: 29 with 2 patients not completing the follow-up). All analyses were conducted based on the per protocol (N = 27) and intention-to-treat population (N = 29).

### 2.4. IRB Approval

The study protocol (LND-0028-25) was approved by the Institutional Review Board/Independent Ethics Committee, and the research was conducted in accordance with the ethical principles of the Declaration of Helsinki. Written informed consent was obtained from all participants, in which voriconazole was administered for the treatment of refractory DLSO.

## 3. Results

### 3.1. Patients Characteristics

Table 1 presents the baseline demographic and clinical characteristics, including disease duration, mycological profile, and treatment duration. Data were collected from medical records of patients diagnosed with DLSO, confirmed by clinical examination and mycological analysis.

The cohort comprised 14 males (48.3%) and 15 females (51.7%), with a mean age of 42.9 years (range: 26–55) for males and 40.8 years (range: 17–63) for females. Disease duration varied, with 6 patients (20.7%) having DLSO for 2–5 years, 12 (41.4%) for 5–10 years, and 11 (38.0%) for more than 10 years, indicating a chronic condition in most cases. Mycological analysis identified *Trichophyton rubrum* as the predominant pathogen in 24/29 patients (82.8%), followed by *Trichophyton mentagrophytes* in 3/29 (10.3%), and mixed infections (*T. rubrum* and *T. mentagrophytes*) in 2/29 (6.9%).

### 3.2. Treatment Response

Clinical improvement was evaluated at 2, 4, 6–9, and 12 months from the start of voriconazole therapy. Efficacy was defined as the combined achievement of clinical cure (≥90% improvement) and mycological cure (negative KOH microscopy and culture) at 12 months (Table 2). Of the 29 patients with distal lateral subungual onychomycosis (DLSO), 27 completed follow-up and were included in the per-protocol analysis. Following a 3-month course of voriconazole, 15/27 patients (55.6%) achieved both clinical and mycological cure by 12 months, while 20/27 patients (74.1%) attained mycological cure (negative KOH and negative culture).

In terms of clinical improvement at successive time points, at 2 months, no patients achieved complete clinical cure (≥90% improvement), 4/27 (13.8%) showed marked improvement (50–89% cure), 8/27 (27.6%) exhibited moderate improvement (26–49% cure), 15/27 (51.7%) had mild improvement (≤25% cure), and 6/27 (20.7%) showed no improvement (Table 2).

By 4 months, 2/27 patients (6.9%) demonstrated marked improvement, 18/27 (62.1%) showed moderate improvement, 6/27 (20.7%) had mild improvement, and 3/27 (10.3%) showed no improvement.

At 6–9 months, 18/27 patients (62.0%) achieved complete clinical cure, while 9/27 (31.0%) exhibited marked improvement.

At 12 months, among the 27 patients who completed follow-up, 18/27 (66.7%, per protocol) achieved complete clinical cure, 7/27 (25.9%, per protocol) showed marked improvement, 1/27 (3.7%) had mild improvement, and 1/27 (3.7%) showed no improvement.

**Figure 1:**
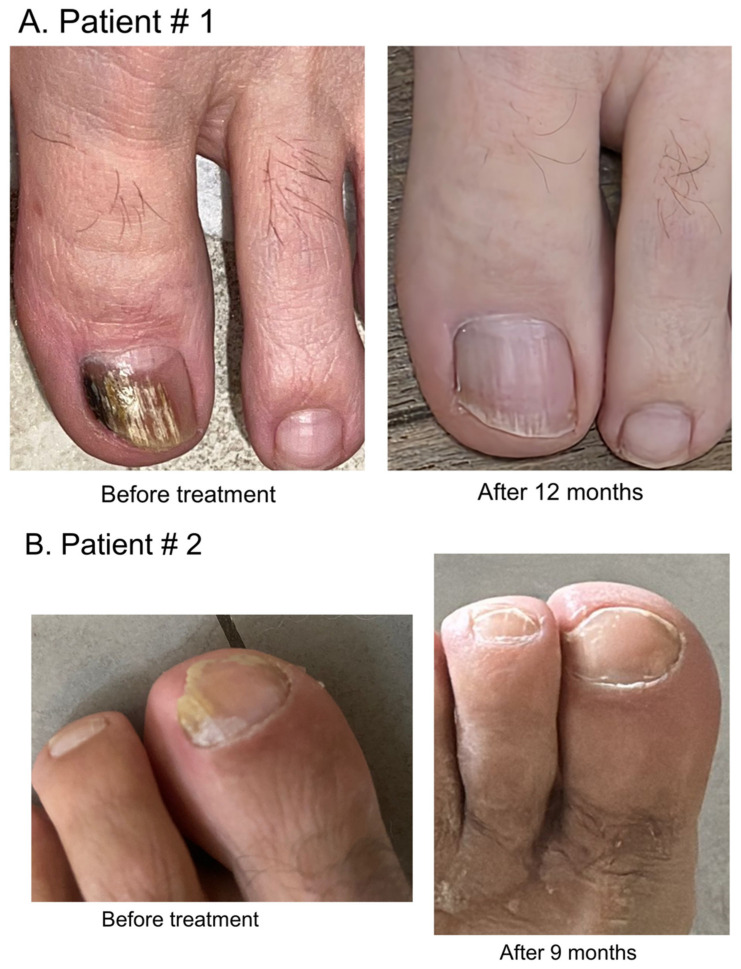

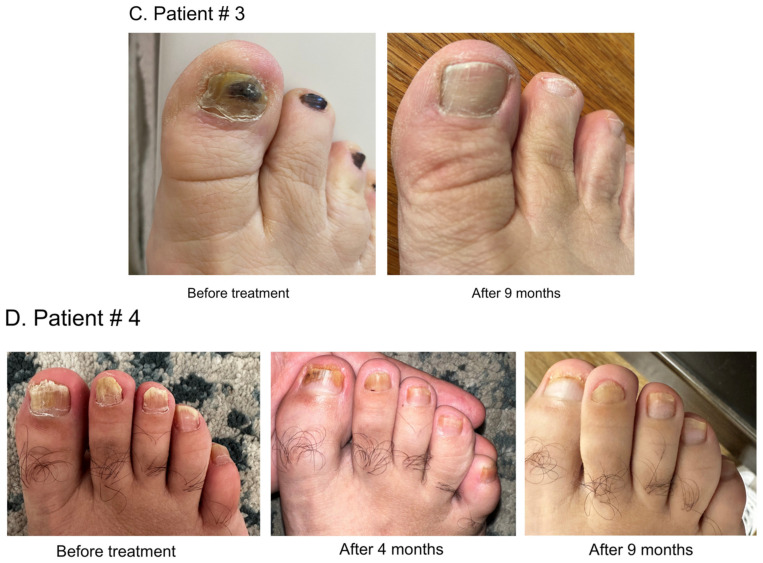
(**A**–**D**) Representative clinical photographs of patients with distal lateral subungual onychomycosis (DLSO) at baseline (prior to treatment with voriconazole) and at 4, 9, and 12 months of follow-up after initiation of voriconazole, demonstrating progressive clinical improvement.

Figure 1 represents clinical photographs of patients with DLSO at baseline and at 4, 9, and 12 months after therapy, demonstrating substantial clinical improvement.

### 3.3. Safety and Tolerability

Liver function tests (LFTs) were conducted and monitored at baseline and at 4, 8, 12, and 16 weeks following initiation of voriconazole (Table 3). In addition, complete blood counts (CBC) and renal function parameters (serum creatinine and urea) were assessed and were normal. At baseline, 26/29 patients (89.7%) had normal LFTs. At week 4, 28/29 patients (96.6%) maintained normal values. At week 8, only one patient (3.4%) showed alanine aminotransferase (ALT) or aspartate aminotransferase (AST) levels more than twice the upper limit of normal (ULN), which normalized without intervention, and 24/29 (82.8%) had normal values. Treatment should be discontinued if transaminases exceed 2.5 × ULN. By week 12, 28/29 patients (96.6%) again had normal results. At week 16, 21/29 patients (72.4%) had normal LFTs, with no cases exceeding twice the ULN.

Patients were monitored for adverse events including visual disturbances, fever, nausea, rash, vomiting, chills, headache, tachycardia, and hallucinations [12]. Only one patient reported mild nausea. No cases of rash, hallucinations, or cardiovascular events were observed.

Given that voriconazole has been associated with visual disturbances [12], patients underwent baseline and follow-up ophthalmologic examinations, including fundoscopy, visual acuity, and visual field testing. No new ophthalmologic abnormalities were detected during the 3–4 month treatment period.

The overall safety profile observed in this cohort aligns with prior case reports of voriconazole use in onychomycosis, where adverse events were generally mild and self-limited [13,14]. In this study, voriconazole administered as 200 mg twice on day 1, followed by 200 mg once daily for 3–4 months, was well tolerated in patients with difficult-to-treat distal lateral subungual onychomycosis.

## 4. Discussion

Clinical evidence for the use of voriconazole in dermatophyte onychomycosis, particularly distal lateral subungual onychomycosis (DLSO), remains scarce. This retrospective case series contributes to the limited literature and provides practical insights for clinicians managing treatment-refractory disease.

Published reports describe isolated cases in which voriconazole achieved clinical and mycological cure following failure of standard therapies. Nofal et al. reported the successful use of voriconazole in a 52-year-old male liver transplant patient with fingernail onychomycosis caused by *Trichophyton rubrum* [13]. The diagnosis was confirmed via KOH microscopy and fungal culture, although minimum inhibitory concentration (MIC) testing was not performed [13]. The patient had previously failed treatment with both itraconazole and terbinafine [13]. Oral voriconazole, administered as 200 mg twice daily for three months, led to resolution of subungual hyperkeratosis and normalization of nail color and thickness. Follow-up over eight months showed sustained improvement, with no recurrence [13]. Side effects including blurred vision, gastrointestinal discomfort, and headache were mild and transient [13].

In another report, a 30-year-old Nigerian woman with *Scytalidium dimidiatum*–associated onychomycosis and accompanying cutaneous infection responded well to voriconazole therapy [14]. She was treated with a loading dose of 400 mg twice daily, followed by a maintenance dose of 200 mg twice daily for three months [14]. The treatment led to complete resolution of nail dystrophy and periungual swelling, with culture-confirmed clearance at 12 weeks. Some residual nail hyperpigmentation persisted, but no active infection remained. To ensure safety and efficacy, serum voriconazole concentrations were monitored throughout treatment. Considering the plasma concentrations obtained at week 2 and the patient’s body weight (75 kg), the maintenance dose was increased to 300 mg twice daily to achieve and sustain therapeutic levels within the target range of 1.0–1.5 mg/L [14].

The limitations of this study include the lack of antifungal susceptibility testing (MIC determination) and SQLE gene analysis, which could have confirmed terbinafine resistance and provided mechanistic context for treatment response. The study was designed primarily to inform clinical decision-making in routine practice; therefore, we focused on patient-centered endpoints (clinical improvement, mycological cure, and complete cure). Nevertheless, future work incorporating standardized susceptibility testing and SQLE analysis will be valuable to correlate resistance markers with outcomes.

Voriconazole is primarily indicated for the treatment of invasive fungal infections [12]. Therefore, antifungal stewardship remains essential when treating onychomycosis. Voriconazole should be reserved for cases where standard treatments have failed. For mild-to-moderate onychomycosis, topical treatments available in the U.S., including efinaconazole, tavaborole, and ciclopirox, may serve as appropriate first-line options [3,15]. In Europe and many parts of Asia and the Middle East amorolfine may be a consideration. In moderate-to-severe cases, oral antifungals such as terbinafine, itraconazole, or fluconazole are generally preferred [3,16].

When treatment fails, clinicians should first evaluate patient adherence and assess for potential drug interactions. It may also be necessary to confirm the clinical diagnosis, as presumptive diagnoses can be inaccurate [3]. Antifungal susceptibility testing and molecular diagnostics, including PCR to identify the causative organism and squalene epoxidase gene mutation testing for terbinafine resistance, can provide valuable guidance for subsequent therapy [3].

If drug resistance is suspected, alternative strategies such as increasing the dose or extending the duration of terbinafine, itraconazole, or fluconazole may be considered [3]. Combination therapy using systemic antifungals and topical agents is another option [3]. When all conventional approaches have been exhausted, newer-generation azoles like voriconazole (off-label use) may be considered, particularly for treatment-resistant onychomycosis.

## 5. Conclusions

In this retrospective case series, voriconazole achieved favorable clinical and mycological cure rates with minimal adverse events in patients DLSO within 12 months of therapy initiation. These findings suggest that voriconazole may be considered as an off-label therapeutic option for confirmed dermatophyte onychomycosis resistant to terbinafine (± itraconazole), provided its use is guided by antifungal stewardship principles and supported by appropriate microbiological confirmation.

## Figures and Tables

**Table 1:** Baseline characteristics of patients with Distal Lateral Subungual Onychomycosis (DLSO), including disease duration, mycological analysis, and duration of voriconazole treatment.

Sex [*n* (%)], N = 29			
Male		Female	
14/29 (48.3%)		15/29 (51.7%)	
Mean age, years (range), N = 29			
Male		Female	
42.9 (26–55)		40.8 (17–63)	
Duration of DLSO [*n* (%)], N = 29			
1–2 years	2–5 years	5–10 years	More than 10 years
-	6/29 (20.7%)	12/29 (41.4%)	11/29 (38.0%)
Mycological analysis [*n* (%)], N = 29			
*Trichophyton rubrum*	*Trichophyton mentagrophytes*	Mixed (*Trichophyton rubrum* and *Trichophyton mentagrophytes*)	
24/29 (82.8%)	3/29 (10.3%)	2/29 (6.9%)	
Comorbidities [*n* (%)], N = 29			
10/29 (34.48%)			
Treatment duration of voriconazole: 3–4 months			

**Table 2:** Clinical response/Efficacy of voriconazole.

	Clinical Improvement					Mycological Cure			Clinical Plus Mycological Cure	
	After 2 months, N = 29 ^a^, n (%)	After 4 months, N = 29 ^a^, n (%)	After 6–9 months, N = 29 ^a^, n (%)	After 12 months, N = 27 ^b^, n (%)		After 12 months, N = 27 ^b^, n (%)			After 12 months, N = 27 ^b^, n (%)	
Complete clinical cure (≥90% cure)	-	-	18 (62.1%)	18 (PP = 66.7%; ITT = 62.1%)		Negative KOH & negative culture	20 (PP = 74.1%; ITT = 69.0%)		≥90% clinical cure, and Negative KOH & negative culture	15 (PP = 55.6%; ITT = 51.7%)
Marked improvement (50–89% cure)	-	2 (6.9%)	9 (31.0%)	7 (PP = 25.9%; ITT = 24.1%)		Positive KOH & negative culture	3 (PP = 11.1%; ITT = 10.3%)		≥90% clinical cure, and positive KOH & negative culture	2 (PP = 7.4%; ITT = 6.9%)
Moderate improvement (26–49% cure)	8 (27.6%)	18 (62.1%)	-	-		Positive KOH & positive culture	3 (PP = 11.1%; ITT = 10.3%)		≥90% clinical cure, and positive KOH & positive culture	1 (PP = 3.7%; ITT = 3.4%)
Mild improvement (up to 25% cure)	15 (51.7%)	6 (20.7%)	1 (3.4%)	1 (PP = 3.7%; ITT = 3.4%)		Negative KOH & positive culture	1 (PP = 3.7%; ITT = 3.45%)		50–89% clinical cure, and Negative KOH & negative culture	5 (PP = 18.5%; ITT = 17.2%)
No improvement	6 (20.7%)	3 (10.3%)	1 (3.4%)	1 (PP = 3.7%; ITT = 3.4%)					50–89% clinical cure, and positive KOH & positive culture	2 (PP = 7.4%; ITT = 6.9%)
									Up to 25% clinical cure and positive KOH & negative culture	1 (PP = 3.7%; ITT = 3.4%)
									Clinically no improvement, and Negative KOH & positive culture	1 (PP = 3.7%; ITT = 3.4%)

^a^ The study initially started with 29 patients. ^b^ Two patients discontinued follow-up. PP: Per Protocol ITT: Intention-To-Treat.

**Table 3:** Liver function tests, N = 29.

	Normal, *n* (%)	More Than Twice From the ULN ^b^, *n* (%)	Blood Tests Were Not Done, *n* (%)
Baseline ^a^	26 (89.7%)	-	3 (10.3%)
After 4 weeks	28 (96.6%)	-	1 (3.4%)
After 8 weeks+/−10 days	24 (82.8%)	1 (3.4%)	4 (13.8%)
After 12 weeks+/−10 days	28 (96.6%)	-	1 (3.4%)
After 16 weeks+/−10 days	21 (72.4%)	-	8 (27.6%)

^a^ Blood test was conducted after 2 weeks. ^b^ Upper limit of normal.

## Data Availability

The original contributions presented in this study are included in the article material. Further inquiries can be directed to the corresponding author.
